# Oxygen Vacancy-Tuned Physical Properties in Perovskite Thin Films with Multiple B-site Valance States

**DOI:** 10.1038/srep46184

**Published:** 2017-04-18

**Authors:** Erik Enriquez, Aiping Chen, Zach Harrell, Paul Dowden, Nicholas Koskelo, Joseph Roback, Marc Janoschek, Chonglin Chen, Quanxi Jia

**Affiliations:** 1 Center for Integrated Nanotechnologies (CINT), Los Alamos National Laboratory, Los Alamos, NM 87545, USA; 2 Department of Physics and Astronomy, University of Texas at San Antonio, San Antonio, TX 78249, USA; 3 Condensed Matter and Magnet Science Group, Los Alamos National Laboratory, Los Alamos, NM 87545, USA; 4 Department of Materials Design and Innovation, University at Buffalo, The State University of New York, Buffalo, NY 14260, USA

## Abstract

Controlling oxygen content in perovskite oxides with ABO_3_ structure is one of most critical steps for tuning their functionality. Notably, there have been tremendous efforts to understand the effect of changes in oxygen content on the properties of perovskite thin films that are not composed of cations with multiple valance states. Here, we study the effect of oxygen vacancies on structural and electrical properties in epitaxial thin films of SrFeO_3−δ_ (SFO), where SFO is a compound with multiple valance states at the B site. Various annealing treatments are used to produce different oxygen contents in the films, which has resulted in significant structural changes in the fully strained SFO films. The out-of-plane lattice parameter and tetragonality increase with decreasing oxygen concentration, indicating the crystal structure is closely related to the oxygen content. Importantly, variation of the oxygen content in the films significantly affects the dielectric properties, leakage conduction mechanisms, and the resistive hysteresis of the materials. These results establish the relationship between oxygen content and structural and functional properties for a range of multivalent transition metal oxides.

The functionality for a range of complex metal oxides is controlled by the interplay between lattice, spin, charge, and orbital degrees of freedom. Experimentally, it has been demonstrated that the oxygen content is one of the key parameters in controlling this interplay and thus significantly influence the physical properties; for example, Mn-O-Mn chains in manganite perovskite oxides determine the ferromagnetism and magnetotransport properties^1^,^2^. In semiconducting oxides, oxygen vacancies usually serve as dopants (since the energy level is above the middle of the bandgap) and impact the electrical behavior. Because the oxygen content in complex metal oxides allows the manipulation of desired physical properties^3^,^4^,^5^,^6^,^7^,^8^, it is a crucial aspect of consideration in the design, synthesis, and application of functional oxide thin films. In perovskite oxides with ABO_3_ structure, oxygen content-driven A-site and B-site vacancies have been reported to significantly alter the physical properties of thin films. There have been tremendous efforts to understand the effect of changes in oxygen content on the properties of perovskite thin films that are not composed of cations with multiple valance states. While oxygen vacancies have been recently been reported to act as desired defects^9^,^10^,^11^,^12^,^13^,^14^,^15^,^16^,^17^, the oxygen content effect in perovskite compounds with multiple valance states at the B site can provide a deeper understanding of the underlying mechanics of the functional properties observed in these materials^18^.

During thin film growth, oxygen content in oxide thin films varies depending on the growth conditions or post-treatments. Three main approaches have been commonly used to accommodate oxygen vacancies: (I) by generating corresponding vacancies in cation sites; (II) by altering the valence state of cations without cation non-stoichiometry; and (III) by incorporating both cation vacancies and change of valence state. Generation of vacancies in cation sites or forming cation-anion vacancy pairs usually occurs in metal oxides without multiple valence states such as ZnO, SrTiO_3_, BaTiO_3_, etc^19^. For example, electronic conduction and superconductivity in SrTiO_3_ as well as ferroelectricity in BaTiO_3_ are influenced by oxygen vacancies induced cation stoichiometry^20^,^21^,^22^. It was reported that the cation stoichiometry is strongly affected by the oxygen pressure during synthesis. In both SrTiO_3_ and BaTiO_3_, lower oxygen pressure during film deposition results in larger out-of-plane lattice parameter^23^.

Altering the valence state of cations without cation non-stoichiometry usually occurs in compounds with multiple valance states, where oxygen vacancies are often charge-compensated by the change of cation valence state^24^. Perovskite oxides with transition metals such as V, Co, Fe, and Mn belong to this category. SrFeO_3−δ_ is an example of this type. The existence of multiple valence states of Fe allows for various stable states of oxygen occupancy in the lattice. Much of the recent attention in SFO thin films stems from the strong dependence of the crystal structure, magnetic and electrical properties of this material on the oxygen content (0 ≤ δ ≤ 0.5). SrFeO_2.5_ can exhibit semiconducting behavior and brownmillerite structure, while cubic perovskite SrFeO_3_ has been shown to exhibit metallic behavior with existence of helical antiferromagnetic-ordered spin structure^25^,^26^,^28^.

The control of vacancy state behavior in compounds with multiple valance states represents great promise for the malleability of multifunctional oxide thin films, but more investigation is still required to establish and understand the underlying mechanisms. Here, we report on the effect of various processing parameters on the oxygen content of SFO thin films and correlate oxygen content to the transport and dielectric properties. Studying the effect of oxygen vacancies in compounds with multiple valance states such as SFO provides an avenue to better understand the relationship between lattice, charge and structure. We propose that the metastable oxygen deficient states of SFO thin films offer an opportunity to accomplish highly tunable electronic properties applicable to a wide range of technological applications that leverage the variability in structural, magnetic and electrical properties of SFO.

## Results and Discussion

SFO thin films with a thickness of 75 nm were deposited by PLD on Nb-doped (0.7% wt) STO (001) substrates (Nb:STO) at 800 °C in three different post-growth annealing conditions. O_2_ and vacuum anneals were performed by holding the films at 600 °C for 1 hour in O_2_ of 500 Torr and <10^−6^ Torr, respectively. The films were then cooled down to room temperature at 5 °C/min at the same oxygen pressure. SFO with no anneal was also investigated, in which the heater was promptly shut off after deposition and films cooled freely in the growth pressure (250 mTorr O_2_) environment.

**Figure 1:**
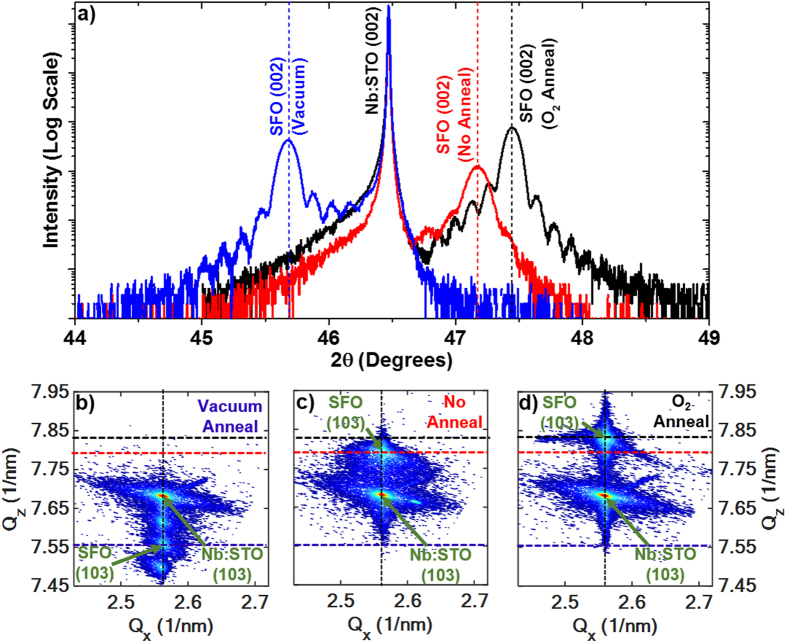
(**a**) 2*θ*-*ω* XRD scan of SFO films grown on Nb:STO (001) substrates annealed in O_2_ (black curve), no anneal (red curve) and vacuum (blue curve). Corresponding RSM scans of (103) SFO film peaks for (**b**) vacuum anneal, (**c**) no anneal and (**d**) O_2_ anneal samples.

Similar to well-studied transition metal oxide systems such as SrCoO_3−δ_^29^, bulk SFO exhibits two topotactic phases: the cubic perovskite SrFeO_3_ and the brownmillerite SrFeO_2.5_^30^,^31^. Perovskite SrFeO_3_ has a bulk lattice parameter of 3.851 Å. The oxygen-deficient brownmillerite SrFeO_2.5_ structure has unit cell parameters of *a* = 5.672 Å, *b* = 15.59 Å, and *c* = 5.527 Å. This structure can be reduced to pseudotetragonal with unit cell parameters of 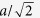
= 4.011 Å, 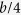 = 3.898 Å, and 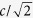 = 3.908 Å^31^,^32^. Figure 1 shows the X-ray diffraction (XRD) patterns of the films treated with different conditions. The out-of-plane lattice parameters of SFO thin films are shown to increase with decreasing oxygen pressure during anneal, as evidenced by the progressively smaller 2*θ* values of the SFO film peak when comparing O_2_ anneal, no anneal and vacuum anneal samples, respectively. A broad XRD 2*θ* scan indicates that films are *c*-axis oriented with no detectable mixed phases present (not shown here). The out-of-plane SFO film lattice parameter is ~3.83 Å for the O_2_ annealed film, ~3.85 Å for the film with no anneal, and ~3.97 Å for the vacuum annealed film. Reciprocal space mapping (RSM) scans around the (103) peak of the SFO films are compared in Fig. 1b–d. In all three films, it can be seen that the in-plane lattice parameter of SFO remains strained to the Nb:STO substrate, indicating *a* = *b* ~3.905 Å. The out-of-plane lattice parameters progressively increase from 3.83 Å to 3.97 Å with reducing oxygen content in annealing. This corresponds to a relative unit cell volume increase of approximately 3.6% when comparing anneal in vacuum *vs*. oxygen. Oxygen vacancies donate electrons to the empty 3*d*-orbitals of Fe, reducing the ion from Fe^4+^ to Fe^3+^ and increasing the ionic radius of the Fe ion^14^. Hence, the lattice parameter is directly related with the oxygen content. These results agree with SFO materials reported by Yamada *et al*.^32^.

**Figure 2:**
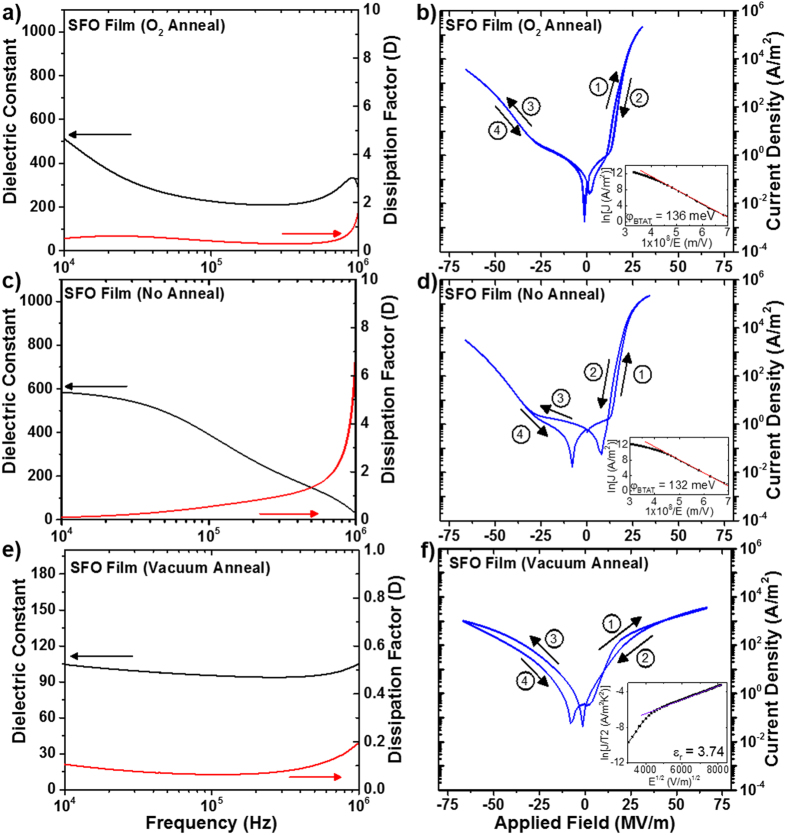
Dielectric constant and loss data, as well as J-V behavior for SFO with O_2_ anneal (**a**,**b**), No anneal (**c**,**d**) and vacuum anneal (**e**,**f**).

The oxygen content and associated structural changes in SFO films significantly modify the electrical properties. The room temperature dielectric and leakage current properties of SFO films with various anneal treatments are shown in Fig. 2. A metal-insulator-metal (MIM) structure was employed with Au top circular electrodes of area approximately 0.3 mm^2^ sputtered on the SFO/Nb:STO samples. In samples with O_2_ anneal and no anneal, the dielectric constant is higher than that of the vacuum annealed samples, but the loss is also significantly greater, with dissipation factor, *D*, of 0.547 and 0.409 for SFO at 100 kHz without anneal and O_2_ annealed, respectively (see Fig. 2a,c and e). The dissipation factor of SFO annealed in vacuum is a magnitude of order lower, with a value of 0.064 at 100 kHz. The lower loss is due to the increased formation of the semiconducting SrFeO_2.5_ brownmillerite phase in the vacuum annealed SFO, which can also be seen in the trend of leakage current behavior in Fig. 2b,d and f. The phase transition between the semiconducting SrFeO_2.5_ phase and metallic SrFeO_3_ phase has been used to design filament type resistive switching devices^31^. The formation of conducting filaments in SFO is possibly related to the migration of oxygen vacancies within SrFeO_3−δ_., under application of electric field, which allows metallic SrFeO_3_ channels to flow current within the semiconducting matrix. As a result, the hysteretic behavior related to this filament formation is directly related to the density and mobility of oxygen vacancies within the SFO bulk. The asymmetry of leakage behavior between positive and negative applied bias is a product of the difference between conduction activity at the Au/SFO and Nb:STO/SFO interfaces^33^,^34^. By applying fields in both directions in the MIM structure, the Schottky diode behavior allows for investigation in the dominant I-V characteristics at either of the metal-semiconductor contact interfaces^35^.

To understand the oxygen vacancies’ effect on electrical properties, the current conduction mechanisms with applied field in the range of 0–66.6 MV/m were analyzed by fitting to both bulk and interface-mediated emission models. Mathematical fitting functions were applied to the I-V curves in regions of applied field above which the majority of hysteresis was observed, since fitting in these regions must also account for the high density of oxygen migration. One possible mechanism of conduction that considers the injection of charge carriers from a metal into nearby oxygen vacancy sites as traps, with an associated barrier height that governs bulk conduction in a solid, is the trap-assisted tunneling current model:^33^


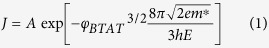


where *A* is a constant, *e* is the electronic charge, 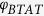 is the corresponding tunneling barrier height, 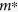 is the effective electron mass, *E* is the electric field magnitude and *h* is the Planck constant. Based on this model, linear fitting was performed on the SFO thin films by plotting *ln*(*J*) *vs.* 1/*E*. A good linear fit was obtained for no anneal and O_2_ annealed SFO samples, suggesting a trap-assisted tunneling conduction mechanism, as seen by the fitting data in the insets of Fig. 2b and d, respectively. The fitting shows that 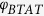 is 132 meV and 136 meV for the SFO samples without annealing and annealed in O_2_, respectively. However, the trap-assisted tunneling model did not yield a good linear trend for the SFO film annealed in vacuum. On the other hand, the Schottky emission model is used to explain temperature-dependent leakage mechanisms that are dominated by injection of charge carriers at the metal-insulator interface, and is given by the following:


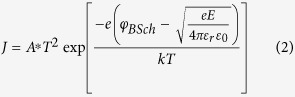


where *J* is the field-dependent current density, *T* is the absolute temperature, 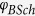 is the Schottky barrier height, 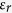 is the optical dielectric constant, 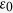 is the permittivity of free space, and *k* is the Boltzmann constant. 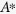 is known as the Richardson constant, represented as


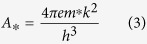


This model also takes into account mediation of leakage current by defects such as oxygen vacancies, which makes it a useful tool to analyze the behavior in SFO thin films^34^,^36^,^37^,^38^. Fitting of the data yields a good linear fit of *ln*(*J*/*T*^2^) *vs*. 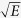 for the vacuum annealed SFO thin film, as can be seen in the inset of Fig. 2f. An optical dielectric constant of 3.74 was extracted from the slope of the linear fitting curve. There are limited data on the optical dielectric constant of SFO, but similar studies on SrCoO_x_ thin films suggest that this value is within reason^39^. Although the results suggest that the leakage current behavior in the vacuum annealed SFO thin film is well-explained by the Schottky equation, the SFO samples without annealing and annealed in O_2_ do not yield a linear trend of the *ln*(*J*/*T*^2^) *vs*. 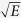 data. Dielectric properties for all SFO films at 100 kHz are compared in Table 1. We can use these data to infer that as the oxygen content in SFO varies, not only the structure but also the nature of the conduction mechanisms in the film undergoes a notable change. Further understanding of the conduction mechanisms is critical to improving and tailoring SFO thin films for specific applications.

**Figure 3:**
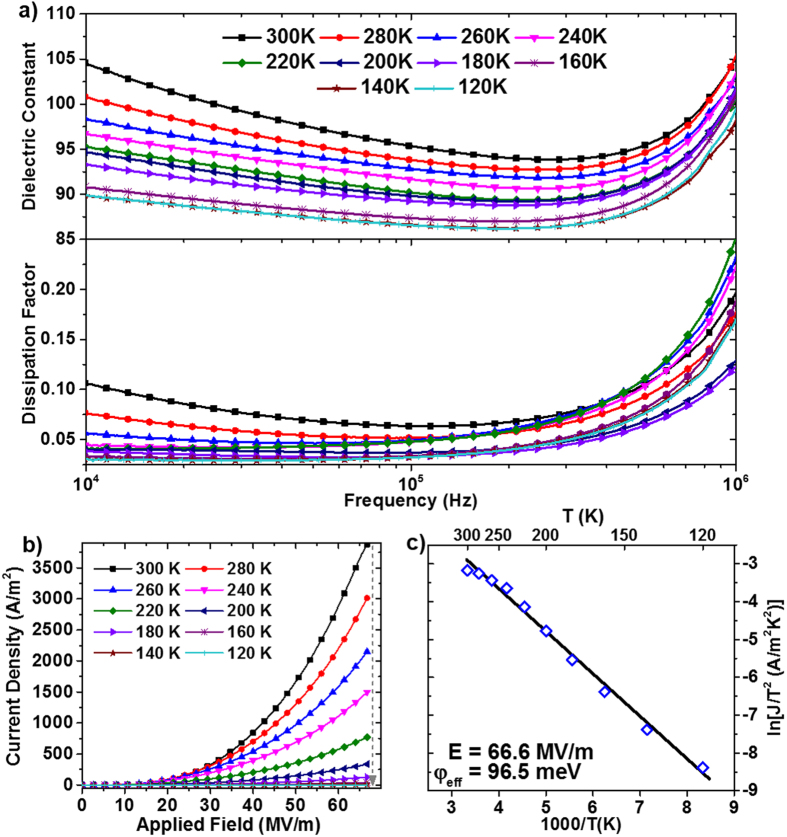
Vacuum annealed SFO temperature dependence in the range of 120 K–300 K of (**a**) Dielectric constant and dissipation factor, (**b**) leakage current *vs*. applied voltage (J-V) behavior with forward bias from 0–66.6 MV/m, (**c**) linear fit of ln(*J*/*T*^2^) *vs*. 1/*T* from which the effective thermionic emission barrier height is calculated.

There are several methods used to extract the Schottky barrier height, 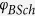, in the Schottky thermionic emission model, but one of the most widely used is to compare the conduction behavior against variation in temperature^38^,^40^. Investigation of the electronic properties of SFO thin film annealed in vacuum was therefore conducted in the temperature range of 120 K–300 K. Results for the temperature dependence of permittivity, dielectric loss and leakage current are shown in Fig. 3.

The dielectric constant and loss of the vacuum annealed SFO sample were observed to decrease with decreasing temperature while maintaining a similar frequency-dependent profile, as shown in Fig. 3a. The temperature dependence of the leakage current at the Au/SFO interface, shown in Fig. 3b, follows an exponential behavior with increasing temperature, which indicates a good fit to the thermionic emission model. An effective thermal barrier height, 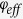, is extracted from the Schottky equation by using the relationship between *ln*(*J*/*T*^2^) *vs. 1/T* at a given *E*, which is presented in Fig. 3c^33^,^38^. By then extrapolating from the linear trend of 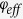 *vs*. 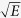 to zero electric field, the intrinsic thermal barrier height, 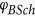, was calculated with a value of 225 meV for the Au/SFO interface.

**Figure 4:**
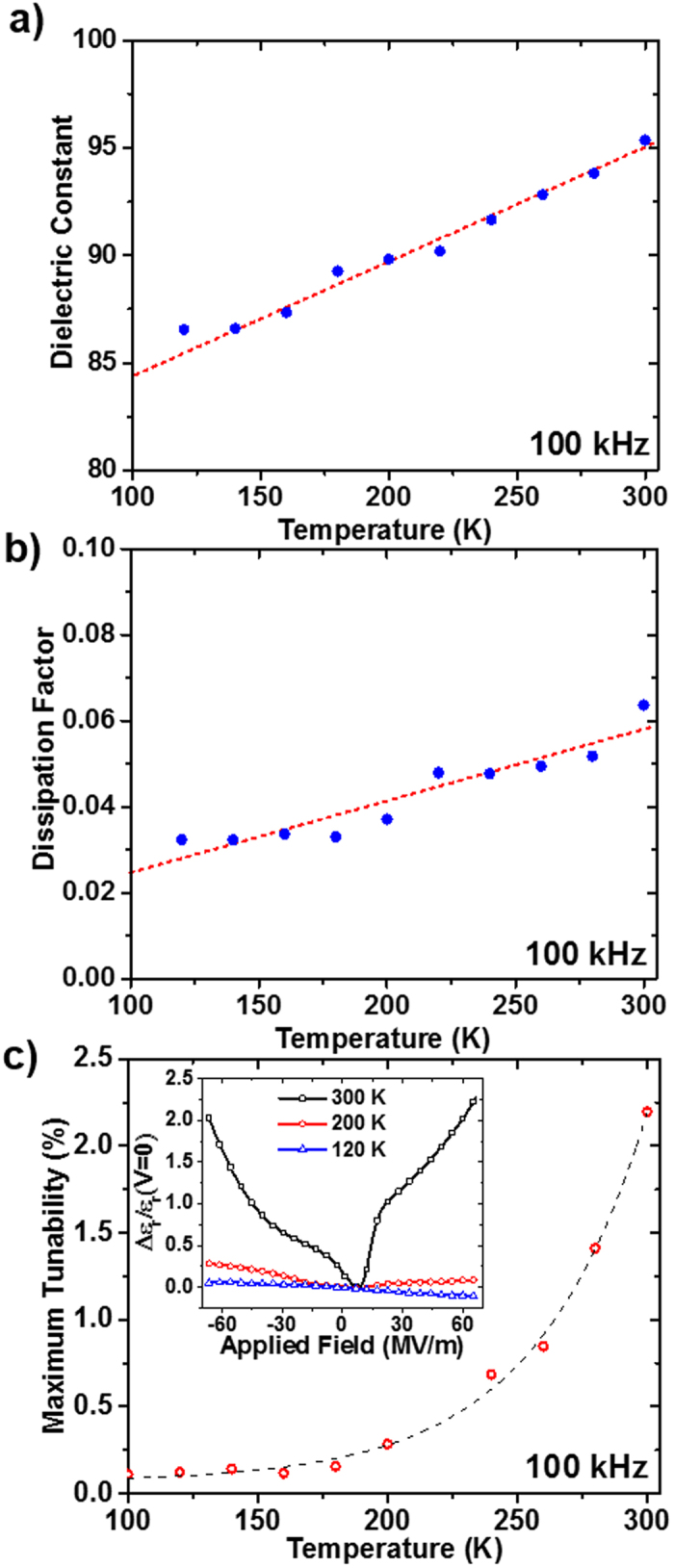
Data at 100 kHz for vacuum annealed SFO *vs*. temperature. (**a**) dielectric constant, (**b**) dissipation factor, (**c**) maximum achievable tunability at an applied field magnitude of 66.6 MV/m, with an exponential dashed fitting curve added as a guide to the eye. Inset shows tunability curves at selected temperatures.

Dielectric property data measured at 100 kHz is summarized in Fig. 4. As temperature is varied, the dielectric response depends on associated changes in volume and polarizability in the dielectric^41^. The linear increasing trend can be attributed to the increasing polarizability of the SFO thin film with temperature, as shown in Fig. 4a and 4b. Indeed, the temperature dependence of the tunability of the SFO thin film, as shown in Fig. 4c, follows an exponential trend in the investigated temperature range. The inset of Fig. 4c shows tunability curves at selected temperatures, where the maximum tunability was calculated at 66.6 MV/m. This behavior is suggestive of the suppression of polarizability in the SFO thin film with decreasing temperature, and indicates that the temperature dependence of polarizability is a dominant effect in governing the response observed in the vacuum annealed SFO thin film^42^. Similar dielectric response behavior has also been seen in LaFeO_3_ in studies reported by Gaikwad *et al*.^43^.

## Conclusion

In summary, the effect of oxygen vacancies on structural and electrical properties in epitaxial thin films of SrFeO_3−δ_ (SFO) is studied, where SFO is a compound with multiple valance states at the B site. Various annealing treatments are used to produce different oxygen contents in the films, which results in significant structural changes in the fully strained SFO films. The out-of-plane lattice parameter and tetragonality increase with decreasing oxygen concentration, indicating the crystal structure is closely related to the oxygen content. Importantly, variation of the oxygen content in the films significantly affects the dielectric properties, leakage conduction mechanisms, and the resistive hysteresis of the materials. Leakage current mechanisms are found to shift from dominantly bulk-mediated trap-assisted tunneling to interface-mediated Schottky thermionic emission depending on oxygen vacancy concentration. Temperature dependence of vacuum annealed SFO thin film is investigated in the temperature range of 120 K–300 K, and results suggested a suppression of polarizability of the SFO thin film with decreasing temperature, as indicated by a reduction in the dielectric constant and tunability of the SFO film. These results establish the relationship between oxygen content and structural and functional properties for a range of multivalent transition metal oxides.

## Methods

SFO thin films were grown by PLD using a KrF excimer laser (Lambda Physik LPX 300, *λ* = 248 nm, 2 Hz). The laser beam, defined by the image beam method^44^, was focused onto the target with an energy density of 1.87 J/cm^2 ^. Prior to the deposition, the chamber was pumped down to a base pressure of 1 × 10^−6^ Torr. A substrate temperature of 800 °C and an oxygen pressure of 250 mTorr were maintained during all depositions. After deposition, various anneal treatments were used to modify the oxygen content in the SFO thin films. An O_2_ anneal was used with an oxygen pressure of 500 Torr and the films were held at 600 °C for 1 hour to allow more oxygen to enter the thin film. The films were then cooled down to room temperature at 5 °C/min. A vacuum anneal was performed by maintaining films in a pressure environment of < 10^−6^ Torr, held at 600 °C followed by cooling to room temperature at 5 °C/min. Samples were also grown with no anneal, in which the heater was promptly shut off after deposition and films cooled freely in the growth pressure (250 mTorr O_2_) environment.

X-ray diffraction (Panalytical X’Pert PRO MRD) 2*θ*-*ω* and reciprocal space mapping (RSM) scans were employed to obtain information on the orientation, lattice parameters and epitaxial quality of the thin films. Dielectric measurements (E4980A Precision LCR meter) were performed in the temperature range of 120 K–300 K by using a physical property measurement system to produce the cryogenic environment. An MIM (Au/SFO/Nb:STO) structure was used for dielectric measurements, with positive bias applied indicating the high potential applied to the Nb:STO substrate and low potential at the Au electrode.

## Additional Information

**How to cite this article:** Enriquez, E. *et al*. Oxygen Vacancy-Tuned Physical Properties in Perovskite Thin Films with Multiple B-site Valance States. *Sci. Rep.* **7**, 46184; doi: 10.1038/srep46184 (2017).

**Publisher's note:** Springer Nature remains neutral with regard to jurisdictional claims in published maps and institutional affiliations.

## Figures and Tables

**Table 1:** Dielectric and leakage conduction mechanism data for SFO films under various anneal conditions.

SFO Post-Growth Anneal	Dielectric Constant at 100 kHz	Dissipation Factor at 100 kHz	Dominant Leakage Mechanism	Barrier Height Calculation (Positive Bias)
Vacuum (PO_2_ < 10^−6^ Torr)	95	0.064	Schottky emission	*φ*_*Bsch*_=225*meV*
No Anneal	391	0.547	Trap-assisted tunneling	*φ*_*BTAT*_=132*meV*
500 Torr O_2_	226	0.409	Trap-assisted tunneling	*φ*_*BTAT*_=136*meV*

## References

[b1] Chen A. P. . Microstructure, Magnetic, and Low-Field Magnetotransport Properties of Self-Assembled (La_0.7_Sr_0.3_MnO_3_)_0.5_:(CeO_2_)_0.5_ Vertically Aligned Nanocomposite Thin Films. Nanotechnology 22, 315712 (2011). 21750374 10.1088/0957-4484/22/31/315712

[b2] Hwang H. Y. . Lattice Effects on the Magnetoresistance in Doped LaMnO_3_, Phys. Rev. Lett. 75, 914 (1995). 10060150 10.1103/PhysRevLett.75.914

[b3] Keeble D. J. . Identification of Vacancy Defects in a Thin Film Perovskite Oxide. Phys. Rev. B 81, 064102 (2010). 10.1103/PhysRevLett.105.226102 21231399

[b4] Xiao G. . High-Temperature Superconductivity in Tetragonal Perovskite Structures - Is Oxygen-Vacancy Order Important. Phys. Rev. Lett. 60, 1446–1449 (1988). 10038040 10.1103/PhysRevLett.60.1446

[b5] Warren W. L., Vanheusden K., Dimos D., Pike G. E. & Tuttle B. A. Oxygen Vacancy Motion in Perovskite Oxides. J. Am. Ceram. Soc. 79, 536–538 (1996).

[b6] Raveau B. & Seikh M. M. in *Cobalt Oxides* 10.1002/9783527645527.ch1 Ch. 1. Crystal Chemistry of Cobalt Oxides, 3–70 (Wiley-VCH Verlag GmbH & Co. KGaA, 2012).

[b7] Anderson M. T., Vaughey J. T. & Poeppelmeier K. R. Structural Similarities Among Oxygen-Deficient Perovskites. Chem. Mater. 5, 151–165 (1993).

[b8] Grenier J. C., Pouchard M. & Hagenmuller P. Vacancy Ordering in Oxygen-Deficient Perovskite-Related Ferrites. Struct. Bond. 47, 1–25 (1981).

[b9] Kalinin S. V. & Spaldin N. A. Functional Ion Defects in Transition Metal Oxides. Science 341, 858–859 (2013). 23970692 10.1126/science.1243098

[b10] Kalinin S. V., Borisevich A. & Fong D. Beyond Condensed Matter Physics on the Nanoscale: The Role of Ionic and Electrochemical Phenomena in the Physical Functionalities of Oxide Materials. ACS Nano 6, 10423–10437 (2012). 23205676 10.1021/nn304930x

[b11] Kim Y. M. . Probing Oxygen Vacancy Concentration and Homogeneity in Solid-Oxide Fuel-Cell Cathode Materials on the Subunit-Cell Level. Nature Mater. 11, 888–894 (2012). 22902896 10.1038/nmat3393

[b12] Biskup N. . Insulating Ferromagnetic LaCoO_3−delta_ Films: A Phase Induced by Ordering of Oxygen Vacancies. Phys. Rev. Lett. 112, 087202 (2014).

[b13] Salluzzo M. . Origin of Interface Magnetism in BiMnO_3_/SrTiO_3_ and LaAlO_3_/SrTiO_3_ Heterostructures. Phys. Rev. Lett. 111, 087204 (2013). 24010471 10.1103/PhysRevLett.111.087204

[b14] Kormondy K. J. . Quasi-Two-Dimensional Electron Gas at the Epitaxial Alumina/SrTiO_3_ Interface: Control of Oxygen Vacancies. J. Appl. Phys. 117, 095303 (2015).

[b15] Liu Z. Q. . Bandgap Control of the Oxygen-Vacancy-Induced Two-Dimensional Electron Gas in SrTiO_3_. Adv. Mater. Interfaces 1, 1400155 (2014).

[b16] Enriquez E. . Oxygen Vacancy-Driven Evolution of Structural and Electrical Properties in SrFeO_3_−δ Thin Films and a Method of Stabilization. Appl. Phys. Lett. 109, 141906 (2016).

[b17] Harrell Z. . Oxygen Content Tailored Magnetic and Electronic Properties in Cobaltite Double Perovskite Thin Films. Appl. Phys. Lett. 110, 093102 (2017).

[b18] Jeen H. . Reversible Redox Reactions in an Epitaxially Stabilized SrCoOx Oxygen Sponge. Nat. Mater. 12, 1057–1063 (2013). 23975056 10.1038/nmat3736

[b19] Lee C. H. . Effect of Stoichiometry on the Dielectric Properties and Soft Mode Behavior of Strained Epitaxial SrTiO_3_ Thin Films on DyScO_3_ Substrates. Appl. Phys. Lett. 102, 082905 (2013).

[b20] Schooley J. F., Hosler W. R. & Cohen M. L. Superconductivity in Semiconducting SrTiO_3_. Phys. Rev. Lett. 12, 474–475 (1964).

[b21] Hou Y. S., Xiang H. J. & Gong X. G. Intrinsic Insulating Ferromagnetism in Manganese Oxide Thin Films. Phys. Rev. B 89, 064415 (2014).

[b22] Lee S. A. . Phase Transitions via Selective Elemental Vacancy Engineering in Complex Oxide Thin Films. Sci. Rep. 6, 23649 (2016). 27033718 10.1038/srep23649 PMC4817049

[b23] Chen A. P. . Strong Oxygen Pressure Dependence of Ferroelectricity in BaTiO_3_/SrRuO_3_/SrTiO_3_ Epitaxial Heterostructures. J. Appl. Phys. 114, 124101 (2013).

[b24] Jeen H. . Reversible Redox Reactions in an Epitaxially Stabilized SrCoOx Oxygen Sponge. Nat. Mater. 12, 1057–1063 (2013). 23975056 10.1038/nmat3736

[b25] Tofield B. C., Greaves C. & Fender B. E. F. SrFeO_2.5_ - SrFeO_3.0_ System - Evidence of a New Phase Sr_4_Fe_4_O_11_ (SrFeO_2.75_). Mater. Res. Bull. 10, 737–746 (1975).

[b26] Adler P. . Magnetoresistance Effects in SrFeO_3−δ_: Dependence on Phase Composition and Relation to Magnetic and Charge Order. Phys. Rev. B 73, 094451 (2006).

[b27] Chakraverty S. . Multiple Helimagnetic Phases and Topological Hall Effect in Epitaxial Thin Films of Pristine and Co-doped SrFeO_3_. Phys. Rev. B 88, 220405 (2013).

[b28] Oda H., Yamaguchi Y., Takei H. & Watanabe H. Single-Crystal Neutron-Diffraction Study of SrFeO_3−x_ (x = 0.1). J. Phys. Soc. Jpn. 42, 101–106 (1977).

[b29] Jeen H. . Topotactic Phase Transformation of the Brownmillerite SrCoO2.5 to the Perovskite SrCoO_3_−δ. Adv. Mater. 25, 3651–3656 (2013). 23852832 10.1002/adma.201300531

[b30] Chakraverty S., Ohtomo A., Okude M., Ueno K. & Kawasaki M. Epitaxial Structure of (001)- and (111)-Oriented Perovskite Ferrate Films Grown by Pulsed-Laser Deposition. Cryst. Growth Des. 10, 1725–1729 (2010). 10.1021/cg901355c PMC2851191 20383295

[b31] Acharya S. K. . Epitaxial Brownmillerite Oxide Thin Films for Reliable Switching Memory. ACS Appl. Mater. Interfaces 8, 7902–7911 (2016). 26955744 10.1021/acsami.6b00647

[b32] Yamada H., Kawasaki M. & Tokura Y. Epitaxial Growth and Valence Control of Strained Perovskite SrFeO_3_ Films. Appl. Phys. Lett. 80, 622–624 (2002).

[b33] Mojarad S. A. . A Comprehensive Study on the Leakage Current Mechanisms of Pt/SrTiO_3_/Pt Capacitor. J. Appl. Phys. 111, 014503 (2012).

[b34] Hudec B. . Atomic Layer Deposition Grown Metal-Insulator-Metal Capacitors with RuO_2_ Electrodes and Al-doped Rutile TiO_2_ Dielectric Layer. J. Vac. Sci. Technol. B 29, 01AC09 (2011).

[b35] Elhadidy H., Sikula J. & Franc J. Symmetrical Current-Voltage Characteristic of a Metal-Semiconductor-Metal Structure of Schottky Contacts and Parameter Retrieval of a CdTe Structure. Semicond. Sci. Technol. 27, 015006 (2012).

[b36] Wang M. T., Wang T. H. & Lee J. Y. M. Electrical Conduction Mechanism in High-Dielectric-Constant ZrO_2_ Thin Films. Microelectron. Reliab. 45, 969–972 (2005).

[b37] Chakraborty S., Bera M. K., Bhattacharya S. & Maiti C. K. Current Conduction Mechanism in TiO_2_ Gate Dielectrics. Microelectron. Eng. 81, 188–193 (2005).

[b38] Lee S. W., Han J. H. & Hwang C. S. Electronic Conduction Mechanism of SrTiO_3_ Thin Film Grown on Ru Electrode by Atomic Layer Deposition. Electrochem. Solid State Lett. 12, G69–G71 (2009).

[b39] Choi W. S. . Reversal of the Lattice Structure in SrCoO_x_ Epitaxial Thin Films Studied by Real-Time Optical Spectroscopy and First-Principles Calculations. Phys. Rev. Lett. 111, 097401 (2013). 24033069 10.1103/PhysRevLett.111.097401

[b40] Jeong H. Y., Lee J. Y., Ryu M. K. & Choi S. Y. Bipolar Resistive Switching in Amorphous Titanium Oxide Thin Film. Phys. Status Solidi RRL 4, 28–30 (2010).

[b41] Havinga E. E. The Temperature Dependence of Dielectric Constants. J. Phys. Chem. Solids 18, 253–255 (1961).

[b42] Tagantsev A. K., Sherman V. O., Astafiev K. F., Venkatesh J. & Setter N. Ferroelectric materials for microwave tunable applications. J. Electroceram. 11, 5–66 (2003).

[b43] Gaikwad V. M., Sheikh J. R. & Acharya S. A. Investigation of Photocatalytic and Dielectric Behavior of LaFeO_3_ Nanoparticles Prepared by Microwave-Assisted Sol-Gel Combustion Route. J. Sol-Gel Sci. Technol. 76, 27–35 (2015).

[b44] Dowden P. C. & Jia Q. X. Stabilizing laser energy density on a target during pulsed laser deposition of thin films. United States patent 9353435 (2016).

